# The Geroprotective Drug Candidate CMS121 Alleviates Diabetes, Liver Inflammation, and Renal Damage in db/db Leptin Receptor Deficient Mice

**DOI:** 10.3390/ijms24076828

**Published:** 2023-04-06

**Authors:** Saadia Zahid, Alcir L. Dafre, Antonio Currais, Jingting Yu, David Schubert, Pamela Maher

**Affiliations:** 1 Cellular Neurobiology Laboratory, Salk Institute for Biological Studies, La Jolla, CA 92037, USA; 2 Neurobiology Research Laboratory, Atta ur Rahman School of Applied Biosciences, National University of Sciences and Technology (NUST), Islamabad 44000, Pakistan; 3 Biochemistry Department, Federal University of Santa Catarina, Florianópolis 88040-900, Brazil; 4 The Razavi Newman Integrative Genomics and Bioinformatics Core, Salk Institute for Biological Studies, La Jolla, CA 92037, USA

**Keywords:** geroneuroprotector, Alzheimer’s disease, obesity, metabolic disorders, inflammation, kidney damage

## Abstract

db/db mice, which lack leptin receptors and exhibit hyperphagia, show disturbances in energy metabolism and are a model of obesity and type 2 diabetes. The geroneuroprotector drug candidate CMS121 has been shown to be effective in animal models of Alzheimer’s disease and aging through the modulation of metabolism. Thus, the hypothesis was that CMS121 could protect db/db mice from metabolic defects and thereby reduce liver inflammation and kidney damage. The mice were treated with CMS121 in their diet for 6 months. No changes were observed in food and oxygen consumption, body mass, or locomotor activity compared to control db/db mice, but a 5% reduction in body weight was noted. Improved glucose tolerance and reduced HbA1c and insulin levels were also seen. Blood and liver triglycerides and free fatty acids decreased. Improved metabolism was supported by lower levels of fatty acid metabolites in the urine. Markers of liver inflammation, including NF-κB, IL-18, caspase 3, and C reactive protein, were lowered by the CMS121 treatment. Urine markers of kidney damage were improved, as evidenced by lower urinary levels of NGAL, clusterin, and albumin. Urine metabolomics studies provided further evidence for kidney protection. Mitochondrial protein markers were elevated in db/db mice, but CMS121 restored the renal levels of NDUFB8, UQCRC2, and VDAC. Overall, long-term CMS121 treatment alleviated metabolic imbalances, liver inflammation, and reduced markers of kidney damage. Thus, this study provides promising evidence for the potential therapeutic use of CMS121 in treating metabolic disorders.

## 1. Introduction

Type 2 diabetes mellitus (T2DM) is on the rise globally, with the World Health Organization predicting 350 million cases by 2030 [1]. This chronic hyperglycemia results in comorbidities, including cardiovascular disease and certain types of cancer, and can lead to life-threatening conditions such as end-stage kidney disease [2,3]. In the US, 26 million people have diabetes, and 80 million are pre-diabetic. There is a strong connection between T2DM and Alzheimer’s disease (AD), with some proposing that T2DM may be a contributing factor to the development of AD [4]. The relationship between dysregulated energy metabolism and neurodegeneration, as well as T2DM’s increased risk of AD, suggests a link between T2DM and the etiology of AD [5]. Compelling recent literature has shown numerous associations between AD and metabolic disease. For example, insulin resistance and impaired glucose metabolism have important roles in the pathophysiology of dementia, cognitive decline, and AD [6]. Mitochondrial dysfunction, a common factor seen in both AD and T2DM, could lead to energy shortages in the hippocampus, potentially explaining the memory impairment commonly found in AD [7]. Oxidative damage to lipids can lead to lipotoxicity, a potential cause of inflammation, insulin resistance, amyloid accumulation, endoplasmic reticulum stress, ferroptosis, and autophagy, which are shared biological events in the pathogenesis of T2DM and AD [8]. In addition, the interplay among oxidative stress, insulin resistance, and brain AMPK signaling may be associated with the neurotoxic events that contribute to neurodegeneration in T2DM and AD [9]. Importantly, the AD drug candidate CMS121 modulates AMPK signaling in the brain, prevents lipotoxicity induced by ferroptosis, and improves cognitive parameters in an animal model of sporadic AD [10,11,12]. However, it is not yet known if CMS121 can improve diabetes-induced metabolic disturbances.

The high cost of medical treatment for T2DM, which reaches hundreds of billions of dollars annually, has a significant impact on global health budgets. In the US, T2DM is a leading cause of death [13,14] and is often accompanied by chronic kidney disease (CKD) [15]. Existing treatments have limited effectiveness, making the development of low-cost, effective drugs to prevent and/or slow T2DM progression a pressing medical challenge. Despite glucose-lowering drugs reducing blood sugar, they provide limited cardiovascular benefits, and often cause weight gain, especially with insulin and sulfonylurea drugs [16,17]. While metformin is a well-known glucose-lowering drug with benefits, it is not recommended for all patients, such as those with certain cancers, and has shown mixed results in terms of progression-free survival, with better outcomes in patients with reproductive cancers and worse outcomes in those with digestive system cancers [18].

Genetics plays a significant role in T2DM development [19], with body weight, height, and abdominal fat as known risk factors [20]. However, environmental factors such as diet also play a major role in the development and reversal of T2DM markers [21,22]. Db/db mice exhibit obesity, hyperglycemia, and insulin resistance, and are considered a good T2DM model [23,24,25]. Animal models, such as db/db mice, which lack the leptin receptor, can be used to test new T2DM treatments. These mice also display increased levels of hepatic lipids, inflammation, and CKD related to oxidative damage and fibrosis, which are all associated with T2DM in humans as well [26,27,28]. CKD is a particularly serious problem associated with T2DM, so recent metabolomic studies have aimed to identify new CKD markers in urine for early diagnosis [29,30,31]. Another relevant aspect is that T2DM is linked to cognitive decline, with patients having a twofold increased all-cause risk of developing dementia, including vascular and AD, and even those without a dementia diagnosis often show cognitive decline and impairment [32,33,34].

The geroneuroprotector drug candidate CMS121 was synthesized based on the strawberry flavonoid fisetin and demonstrated improved pharmacological properties and efficacy in vitro and in vivo [35,36,37]. A new drug discovery platform based on oxytosis/ferroptosis was used to identify novel neuroprotective compounds, identifying CMS121 as a drug candidate for age-associated diseases, including AD [10]. CMS121 has been successfully employed in animal models of AD and aging, such as in age-accelerated SAMP8 mice [11,38,39]. Given that fisetin, the CMS121 precursor, showed beneficial effects in a mouse model of type 1 diabetes [40], that age is a major risk factor for the development of T2DM, and that CMS121 can reduce markers of age-related CKD in SAMP8 mice [39], we hypothesized that CMS121 might be effective in alleviating the major disturbances observed in db/db mice. To test this hypothesis, young db/db mice were administered a diet containing CMS121 for 6 months. This treatment regimen resulted in significant protection on specific endpoints related to hyperglycemia, liver inflammation, and kidney damage.

## 2. Results

The anti-inflammatory and other protective effects of CMS121 in animal models of aging [11,39] and AD [38] prompted us to search for beneficial effects in db/db mice, a model of T2DM [25,41]. The characteristics of this model include, beyond the diabetes phenotype, memory impairment [42,43], increased lipid levels and hepatic inflammation [26,27,28,44], and CKD [45]. We investigated whether a diet containing CMS121 for 6 months can improve glucose metabolism, lipid status, liver inflammation, and CKD. We also evaluated mitochondrial markers in the liver and kidney, as well as performed a metabolomic analysis of the urine.

### 2.1. Food Intake, Weight Gain, Body Mass, Locomotion, Oxygen Consumption, and Memory

**Figure 1:**
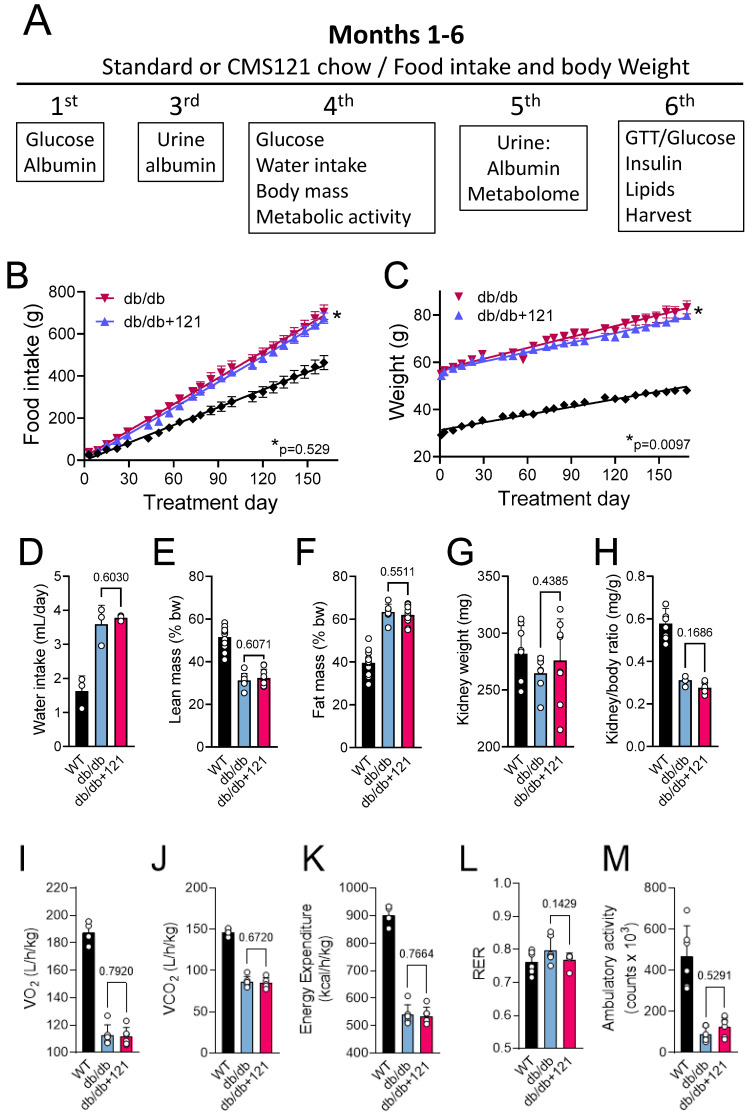
Experimental timeline and nutritional parameters, body mass, locomotor activity, and metabolic activity of db/db mice fed a control diet or a diet containing the AD drug candidate CMS121 for 6 months. Beginning at the 5th week after birth, mice were either kept on the control diet (db/db) or on a diet containing CMS121 (db/db + 121) ad libitum (See M&M for details). (**A**) Timeline and end points analyzed. Wildtype (WT) mouse data are presented as a reference. Cumulative food intake (**B**) and body weight (**C**). Food intake per animal was based on the food consumption in cages containing 3 mice. Data (**B**,**C**) are presented as average ± SEM (n = 8–12). Differences between linear regression slopes of control and CMS121-treated db/db mice were analyzed, and the *p*-values are indicated. During the 13th and 15th weeks, respectively, treatment, water intake (n = 3), and body mass (n = 8–9) indexes were obtained: (**D**) water intake; (**E**) lean mass, and (**F**) fat mass. Kidney weight (**G**), and kidney/body weight ratio (**H**) data were obtained from 7–8 animals per group at the end of the experiment. Metabolic activity ((**I**–**M**), n = 6) was evaluated at the 15th week of treatment. (**I**) Oxygen consumption (VO_2_); (**J**) carbon dioxide production (VCO_2_); (**K**) energy expenditure; (**L**) respiratory exchange rate (RER); and (**M**) overall ambulatory activity. Data (**D**–**M**) are presented as mean ± SD, and *p*-values are indicated for the untreated control db/db mice as compared to the CMS121-treated mice. Values of the WT group are presented as a reference. One-way ANOVA was used to detect mean changes, and differences between db/db and db/db + 121 were evaluated by the Holm–Sidak post-hoc test.

Figure 1A presents the experimental timeline. Some basic comparisons between the db/db mice on the control and CMS121 diets, including food intake, body weight, and mass indices, are shown in Figure 1B–M. The elevated food intake (Figure 1B) is known to cause weight gain in db/db mice (Figure 1C). At the end of the 6-month treatment, db/db mice were 1.7-fold heavier compared to wildtype (WT) mice. Higher water intake, as observed for control db/db mice (Figure 1D) compared to the WT group, was also seen in diabetes [46]. The db/db mice receiving CMS121 in the diet had a similar food intake compared to mice receiving the standard diet (Figure 1B). Despite the similar food intake, db/db mice treated with CMS121 showed a significantly lower (5%), but still substantial weight gain (Figure 1C), while water intake was similar (Figure 1D). Body composition was altered by the db/db genotype (Figure 1E,F) and was not changed by the CMS121 diet, as observed by similar average lean (Figure 1E) and fat (Figure 1F) masses. The CMS121 diet also did not affect the kidney weight (Figure 1G) or the kidney weight/body weight ratio (Figure 1H), which both decreased in the db/db mice relative to WT mice.

Metabolic parameters of the control db/db mice included lower VO_2_ (Figure 1I), VCO_2_ (Figure 1J), and energy expenditure (Figure 1K) relative to WT mice, without significant changes in the respiratory exchange rate (Figure 1L). The CMS121 diet did not alter any of these metabolic parameters (Figure 1I–L). Locomotor activity was also lower in the db/db mice (Figure 1M) as compared to the WT mice and was not restored by the CMS121 diet.

### 2.2. Glucose and Lipid Status

**Figure 2:**
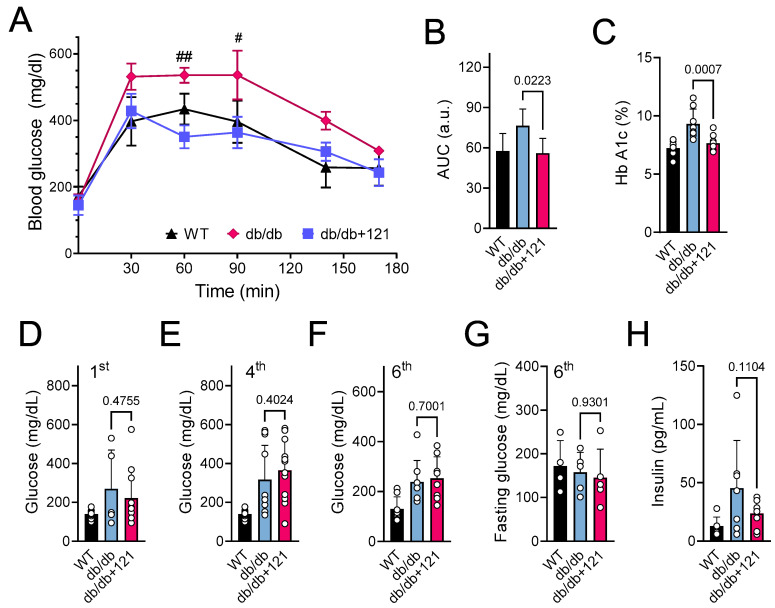
Glucose status in db/db mice that were untreated or treated with CMS121 for 6 months. (**A**) Glucose tolerance test (GTT, n = 4–5) and (**B**) area under the curve (AUC) were obtained at the 6th month of treatment. (**C**) At the end of the experiment, blood was collected for the measurement of hemoglobin A1c (HbA1c; n = 8–9). Glucose was evaluated by caudal vein puncture (n = 11–12) after the 1st (**D**), 4th (**E**), and 6th ((**F**), n = 8) month of treatment. Fasting glucose was also evaluated in animals at 6 months of treatment ((**G**), n = 4–5). (**H**) Insulin levels were evaluated at the end of the treatment (n = 6–8). Data are presented as mean ± SD, except for GTT (mean ± SEM). *p*-values are indicated for the untreated control db/db mice compared to the CMS121-treated mice. Values of WT mice are presented as a reference. For the GTT (**A**), two-way ANOVA was used followed by the Bonferroni post-hoc test for multiple comparison analysis. One-way ANOVA was used to detect mean changes, and differences between db/db and db/db + 121 were evaluated by the Holm–Sidak post-hoc test (**B**–**H**). ^#^ *p* < 0.05 and ^##^ *p* < 0.01 as compared to WT at the same timepoint.

Control db/db mice presented a decreased ability to move glucose out of the blood in the glucose tolerance test (GTT) (Figure 2A,B), as well as increased glucose levels (Figure 2D–F), compared to the WT mice. In addition to increased levels of glucose, db/db mice also showed increased levels of HbA1C (Figure 2C) and elevated levels of plasma insulin (Figure 2H), two hallmarks of diabetes. We found that the CMS121 diet induced a significantly better outcome in the GTT (Figure 2A,B) and lower levels of HbA1c (Figure 2C). A strong trend to lower plasma insulin levels was also observed (Figure 2H). Despite these improvements in the diabetes status, non-fasting glucose levels remained elevated during treatment with the CMS121 diet (Figure 2D–F) compared to the WT mice. Curiously, by the end of the treatment, the fasting levels of glucose were similar between WT, control db/db mice, and db/db mice treated with CMS121 (Figure 2G).

**Figure 3:**
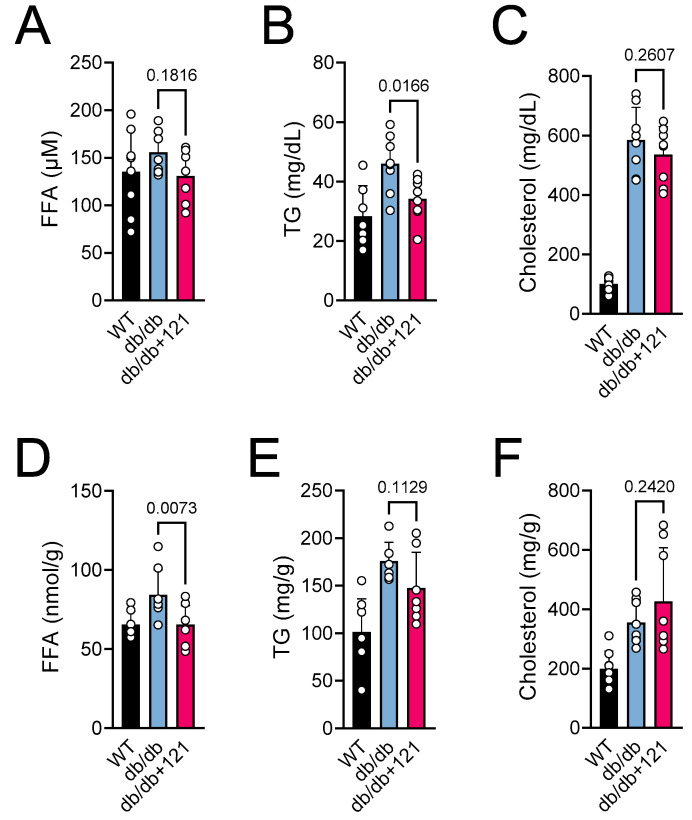
Blood and liver lipids of db/db mice fed control diet or a diet containing CMS121. (**A**,**D**) Free fatty acids (FFA), (**B**,**E**) triglycerides (TG), and (**C**,**F**) cholesterol were evaluated in plasma (**A**–**C**) and liver (**D**–**F**) at the end of the treatment. Data are presented as mean ± SD (n = 7–8). *p*-values are indicated for the untreated db/db mice compared to the CMS121-treated mice. Values of WT mice are presented as a reference. One-way ANOVA was used to detect mean changes, and differences between db/db and db/db + 121 were evaluated by the Holm–Sidak post-hoc test.

An increase in lipids, including free fatty acids (FFA) and triglycerides (TG), is a characteristic of db/db mice, and is recognized to play a central role in the development of T2DM [47,48]. We also observed elevated levels of lipids in plasma (Figure 3A–C) and FFA in liver (Figure 3D–F) in the db/db mice. Control db/db mice presented elevated levels of FFA in the blood (Figure 3A) and liver (Figure 3D), as well as TG (Figure 3B,E), and cholesterol (Figure 3C,F) in both tissues. The CMS121 diet improved the lipid levels in plasma and liver. A tendency for lower levels of FFA was observed in the plasma (Figure 3A), and a significant effect was seen in the liver (Figure 3D). The effect of CMS121 on TG was similar, with a significant decrease in the plasma (Figure 3B), and a tendency to lower levels in the liver (Figure 3E). However, the elevated levels of cholesterol in the blood (Figure 3C) and liver (Figure 3F) of control db/db mice were not altered by CMS121 in the diet.

### 2.3. Liver Inflammation

**Figure 4:**
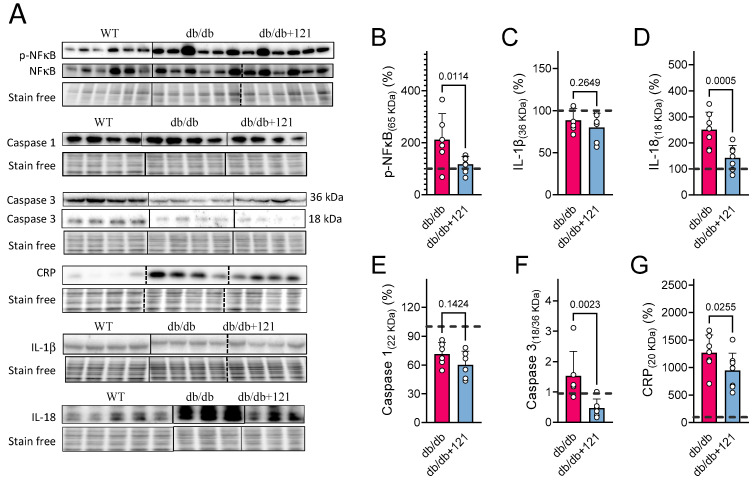
Liver inflammatory markers in db/db mice fed control diet or diet with CMS121. Representative blot images (**A**). Vertical lines indicate non-adjacent lanes from the same blot, and dashed lines adjacent groups. (**B**–**G**) Respective quantification of blots: (**B**) p-NF-κB; (**C**) IL-1β; (**D**) IL-18; (**E**) Caspase 1; (**F**) Caspase 3 (cleaved/uncleaved), and (**G**) C-reactive protein (CRP). Data are presented as mean ± SD (n = 6–8), and *p*-values are indicated for the untreated control db/db mice compared to the CMS121-treated mice. Values were normalized to WT group (dashed line), and data are presented as percentages of WT values. One-way ANOVA was used to detect mean changes, and differences between db/db and db/db + 121 were evaluated by the Holm–Sidak post-hoc test.

Given the increases in lipids in the livers of db/db mice, the reported pro-inflammatory role of lipids [49], and the fact that hepatic inflammation is a known characteristic of db/db mice [27,44], we next evaluated inflammatory markers in the liver using Western blotting (Figure 4A). The quantitative results are presented in Figure 4B–G. Liver inflammation was inferred from elevated levels of active NF-κB (phosphorylated) in the nucleus (Figure 4B) as well as increased levels of IL-18 (Figure 4D) and C-reactive protein (CRP) (Figure 4G). In contrast, no changes in IL-1β (Figure 4C) or caspase 1 (Figure 4E) levels were seen. Cleaved caspase 3 (p18)/caspase 3 ratio (Figure 4F) was also increased in the control db/db mouse livers, indicating higher overall activity of this apoptotic effector protein [50]. The CMS121 diet produced a significant improvement in the hepatic inflammatory status, as observed by lower levels of active NF-κB in the nucleus (Figure 4B), decreased levels of IL-18 (Figure 4D), and CRP (Figure 4G), as well as decreased caspase 3 activity (Figure 4F). IL-1β (Figure 4C) and caspase 1 (Figure 4E) remained below control levels and were not altered by the CMS121 diet.

### 2.4. Kidney Markers

**Figure 5:**
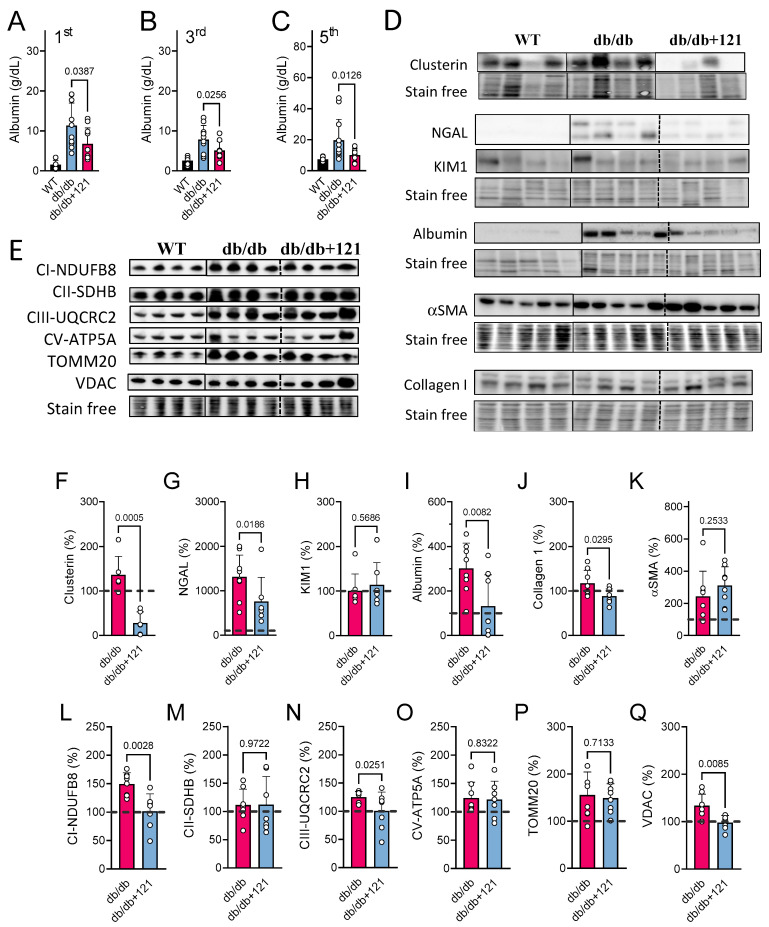
Kidney markers were evaluated in db/db mice fed with CMS121. Urinary albumin levels (**A**–**C**) were evaluated at the 1st, 3rd, and 5th treatment months. Values of control mice (WT) are presented as a reference. Representative blot images of kidney damage markers are presented (**D**) along their respective quantifications: (**F**) clusterin; (**G**) NGAL; (**H**) KIM1; (**I**) albumin, (**J**) collagen; (**K**) α-smooth muscle actin (αSMA). Data are presented as mean ± SD (n = 8–12). Representative blot image (**E**) of kidney mitochondrial proteins and their respective quantifications: markers of mitochondrial complex I ((**L**); NDUFB8), complex II ((**M**); SDHB), complex III ((**N**); UQCRC2), and complex V ((**O**); ATP5A), as well as the outer membrane translocase TOMM20 (**P**), and the voltage-dependent anion channel VDAC (**Q**). Vertical lines indicate non-adjacent lanes from the same blot, and dashed lines adjacent groups. Data are presented as mean ± SD (n = 6–8). *p*-values are indicated for the untreated db/db mice as compared to the CMS121-treated mice. Values were normalized to WT group (dashed line), and data are presented as percentages of WT values. One-way ANOVA was used to detect mean changes, and differences between db/db and db/db + 121 were evaluated by the Holm–Sidak post-hoc test.

Since CKD is a characteristic of disease progression in db/db mice [51,52], we also explored the effects of CMS121 on the kidney. Kidney function was affected in the db/db mice, as evidenced by urinary albuminuria that was increased in the first, third, and fifth experimental months (Figure 5A–C). The CMS121 diet significantly attenuated albuminuria at all time points. Furthermore, at the end of the experiment, protein was extracted from urinary samples and tested by Western blotting. The elevated urinary excretion of albumin found by the ELISA assay (Figure 5A–C) was confirmed in Western blots (Figure 5D,I). Given that in the Western blot, albumin was normalized to total protein in the urine, it confirms the albuminuria found by the ELISA assay (Figure 5A–C).

To provide additional evidence that the CMS121 diet had beneficial effects on the kidneys of db/db mice, we examined additional kidney damage markers in the protein extract of urine and in kidney samples (Figure 5D,F–K). Higher levels of urinary clusterin (Figure 5F) and NGAL (Figure 5G) in the control db/db mice are consistent with an impairment in kidney filtration. KIM1 (Figure 5H), a marker of proximal kidney damage, was not altered in the control db/db mice, compared to the WT control. The CMS121 diet was effective at preventing the increase in clusterin (Figure 5F) and NGAL (Figure 5G) in the urine samples, while urinary KIM1 remained unaltered (Figure 5H).

We also evaluated two markers of kidney fibrosis, collagen I (Figure 5J) and αSMA (Figure 5K). The levels of both proteins were increased in the control db/db kidneys. Collagen I levels were decreased by the CMS121 diet, but αSMA remained elevated relative to WT mouse kidneys.

To further identify markers of altered kidney function, we examined mitochondrial proteins (Figure 5E,L–Q), as it has been shown that db/db mice present altered mitochondrial protein markers in the kidneys [53]. We found that, except for SDHB (Figure 5M), the other five proteins evaluated were altered in control db/db mice, as compared to the WT mice, including NDUFB8 (Figure 5L, *p* = 0.002), UQCRC2 (Figure 5N, *p* < 0.0001), ATP5A (Figure 5O, *p* = 0.045), TOMM20 (Figure 5P, *p* = 0.012), and VDAC (Figure 5Q, *p* = 0.037). The CMS121 diet restored the levels of some of these mitochondrial proteins to a level similar to that seen in WT mice, including markers of the electron transport chain (ETC), such as NDUFB8 (Figure 5L) and UQCRC2 (Figure 5N), and the outer membrane protein VDAC (Figure 5Q). The levels of ATP5A (Figure 5O) and TOMM20 (Figure 5P) were not altered by the CMS121 diet and remained elevated compared to the WT mice.

### 2.5. Urine Metabolome

**Figure 6:**
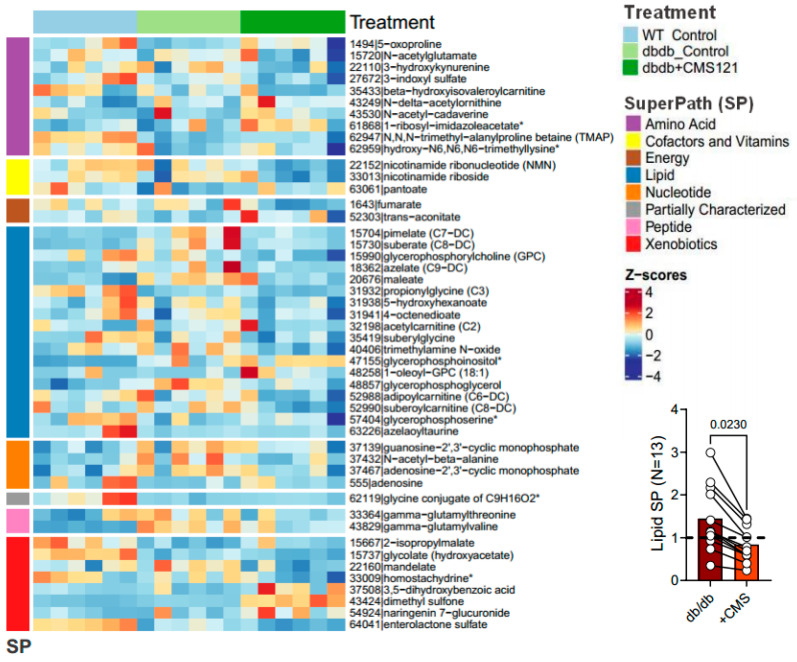
Heatmap of the Z-scores of CMS121-induced changes in control and CMS-treated db/db mouse urine metabolites. Graph insert depicts the metabolites related to fatty acid metabolism that were altered by the diet. The glycine conjugate (C_9_H_16_O_2_) is a partially characterized molecule and was not considered in the analysis. * Indicates compounds that have not been confirmed based on a standard, but mass spectra data was appropriate to reveal its identity.

In recent years, urine has gained more attention as a potential readily accessible, non-invasive source of disease biomarkers. Therefore, we conducted a non-targeted metabolomic study of the urine of the WT, control db/db, and CMS121 diet-fed db/db mice (Figure 6, Supplementary Material S1). In this section, the results are presented for altered metabolite levels with previously established functional relevance.

Our analysis found that 47 metabolites were altered in the urine of the db/db mice that received the CMS121 diet compared to control db/db mice. These alterations involved seven super pathways: amino acids (10), cofactors and vitamins (3), energy (2), lipids (18), nucleotides (4), peptides (2), and xenobiotics (8). Of these, control db/db mice showed 23 changes compared to WT mice. The CMS121 diet modified 16 out of these 23 alterations, tending to restore the values to WT levels. Excluding membrane-derived metabolites, control db/db mice showed an increase in multiple lipids, but the CMS121 diet decreased all 13 fatty acid intermediates in urine (Figure 6, graphic insert). In addition, several gut microbiota-derived metabolites were altered in the urine of the control db/db mice compared to WT mice. The CMS121 diet also produced some changes in microbiota-derived metabolites, including pantoate, 2-isopropylmalic acid, 3,5-dihydroxybenzoic acid, homostachydrine, trimethylamine N-oxide, N-acetylcadaverine, trans-aconitate, dimethyl sulfone, naringenin 7-glucuronide, enterolactone, and mandelate. These changes may indicate specific effects of CMS121 on gut microbiota composition, but further research is needed to determine their functional relevance and potential benefits for alleviating the db/db phenotype.

#### 2.5.1. Metabolomic Changes Associated with Fuel/Energy Metabolism

The CMS121 diet also caused decreases in urinary levels of suberylglycine and propionylglycine, byproducts of fatty acid metabolism whose levels reflect how that metabolism is activated or diminished [54,55]. In addition, the CMS121 diet decreased the four acylcarnitines identified (acetylcarnitine (C2), β-hydroxyisovaleroylcarnitine (C5), adipoylcarnitine (C6-DC), and suberoylcarnitine (C8-DC)) in urine. The diet induced decreases in suberylglycine and propionylglycine, as well as acylcarnitine, suggest that CMS121 induces a decrease in fatty acid metabolism.

Increased levels of dicarboxylic acids in urine have been linked to starvation, high ketones in diabetes, and high-fat diets [56,57,58,59]. The control db/db mice had elevated levels of maleate, pimelate, azelate, and suberate compared to WT mice, consistent with diabetes. The CMS121 diet resulted in a significant decrease in all four dicarboxylic acids and also reduced the levels of 4-octenedioate and 5-hydroxyhexanoate, two medium-chain fatty acids that were not altered in control db/db mice. Together, these changes further point to a lipid-lowering effect of the CMS121 diet.

N-acetyl-β-alanine, a derivative of alanine, can be converted into acetyl-CoA, thus, fueling the tricarboxylic acid cycle [60]. Control db/db mice showed an increase in N-acetyl-β-alanine compared to WT animals. The CMS121 diet decreased N-acetyl-β-alanine, as compared to the control db/db mice, suggesting a decrease in tricarboxylic acid cycle activity. Additionally, the CMS121 diet restored hydroxy-N6,N6,N6-trimethyllysine levels, a key precursor for carnitine synthesis, which was depleted in the db/db mouse urine, and which is thought to facilitate acyl-carnitine turnover. Given that we have only limited information, and only from urine metabolites, the exact role of the CMS121 diet on the tricarboxylic acid cycle remains to be investigated.

#### 2.5.2. Metabolomic Changes Associated with Kidney Dysfunction

In chronic renal failure, tryptophan degradation by the kynurenine pathway is drastically enhanced, leading to an increased burden of toxins reaching the kidney, which are eventually excreted in urine [61]. A high-fat diet also raises indoxyl sulfate in urine [62]. Our study found that the CMS121 diet decreased the urinary levels of indoxyl sulfate and 3-hydroxykynurenine, two tryptophan metabolites, further indications of a lower toxic burden on the kidneys.

5-oxoproline (pyroglutamic acid) and glutamyl-peptides showed an inverse relationship with kidney failure with replacement therapy [63,64,65]. In this study, control db/db mouse urine had lower urinary 5-oxoproline concentrations, which were restored to WT levels by the CMS121 diet, also suggesting a protective mechanism.

Higher glycolate levels in urine are positively associated with kidney function, while lower levels are correlated with kidney damage in T2DM [66,67]. Control db/db mouse urine showed a marked decrease in glycolic acid levels (an indicator of kidney damage) compared to WT mice, and the CMS121 diet partially reversed this decrease.

Elevated levels of N-acetylalanine in serum have been linked to impaired kidney function and modestly associated with all-cause mortality [65,68]. N-acetyl alanine levels were increased in the control db/db mouse and decreased by the CMS121 diet, further supporting a possible beneficial effect on kidney function.

A 10-fold decrease in the mean urinary adenosine levels was observed in the control db/db mice, compared to WT mouse urine. The CMS121 diet produced a 5.5-fold increase in urinary adenosine levels, which amounts, on average, to 50% of WT levels. Adenosine is believed to offer protection to the kidneys [69,70]. Furthermore, under stress, kidneys release adenosine 2′,3′-cyclic monophosphate (2′,3′-cAMP) and 2′,3′-cGMP-guanosine (2′,3′-cGMP) [71]. The increased levels of 2′,3′-cAMP and 2′,3′-cGMP in the db/db mouse urine were reduced towards WT levels by CMS121. These changes also point to a protective mechanism of CMS121 on purine metabolism.

The accumulation of uremic toxins, such as trans-aconitate, is possibly due to a modified microbiota. Additionally, trans-aconitate has been suggested to be involved in the progression of CKD [72]. While the CMS121 diet produced an increase in trans-aconitate in the db/db mouse urine, as compared to control db/db mice, the values were not statistically different from WT levels.

**Figure 7:**
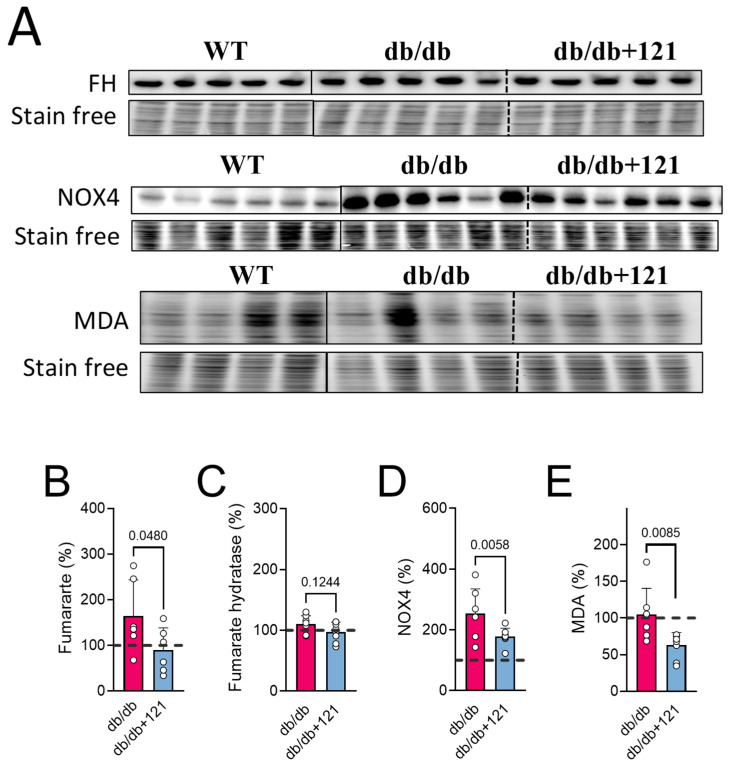
NOX4, MDA, and fumarate are modulated by the CMS121 diet. (**A**) Representative blot images of kidney extracts and their quantification: (**C**) fumarate hydratase (FH), (**D**) NOX4, and (**E**) MDA. (**B**) Fumarate levels in urine obtained from the metabolomic study (Figure 6). Vertical lines indicate non-adjacent lanes from the same blot, and dashed lines adjacent groups. Data are presented as mean ± SD (n = 6–8). *p*-values are indicated for the untreated db/db mice compared to the CMS121-treated mice. Values were normalized to WT group (dashed line), and data are presented as percentages of WT values. One-way ANOVA was used to detect mean changes, and differences between db/db and db/db + 121 were evaluated by the Holm–Sidak post-hoc test.

The main function of NADPH oxidase is to catalyze the formation of reactive oxygen species, and renal NOX4 expression has been shown to be increased in podocytes and mesangial cells in the course of diabetes, contributing to CKD [73]. NOX4 was also associated with fumarate metabolism [74]. The observation of increased levels of fumarate in the urine of the control db/db mice (Figure 6 and Figure 7B) and the finding that CMS121 decreased those levels, prompted us to investigate if NOX4 and fumarate hydratase (FH) levels in the kidneys were altered by the CMS121 diet. We further investigated if MDA, a final product of lipid peroxidation, was also altered in the kidney by the diet. Representative blot images of FH, NOX4, and MDA are presented in Figure 7A. The expression of FH remained at WT levels in the db/db mouse kidneys (Figure 7C) and was not altered by the CMS121 diet. Increased levels of renal NOX4 were observed in the control db/db mouse kidneys (Figure 7D) compared to WT mice, while MDA levels were not altered in the db/db mouse kidneys (Figure 7E), compared to WT levels. Interestingly, the CMS121 diet significantly decreased the expression of NOX4 (Figure 7D) and MDA (Figure 7E) in the kidney tissues, compared to control db/db mice and, in the case of MDA, WT mice as well, suggesting a strong anti-oxidant effect of the diet in this tissue.

## 3. Discussion

The db/db mouse is widely used to study T2DM but has faced criticism for its lack of translational validity as leptin receptor deficiency is rare in humans, and the model primarily results from hyperphagia [25,44]. Another challenge of this model is the impact of feeding suppressors on the diabetic profile [75,76,77]. When a drug decreases food intake, then usually the pathology markers are improved, making it hard to interpret the results. Importantly, CMS121 treatment did not affect food consumption but did lead to a small but significant (~5%) reduction in body weight, indicating its benefits are not directly tied to reduced food intake, but may be partially related to somewhat less severe obesity.

### 3.1. Fuel and Energy Metabolism

The db/db mice showed no alterations in metabolic activity with the CMS121 diet, as indicated by unchanged food consumption, locomotor activity, and oxygen consumption. However, their body weight decreased by 5%, and glucose and lipid metabolism improved. Mice on the CMS121 diet showed lower levels of TG and FFA in both plasma and liver, which was corroborated by the urine metabolome, given that CMS121 restored urinary levels of lipid precursors and reduced FFA metabolites. These changes, along with improved GTT and lower HbA1c and insulin levels, suggest significant changes in the metabolic state of the mice brought about by the CMS121 diet. Indeed, this study is the first to demonstrate the efficacy of CMS121 in improving glucose homeostasis in a model of T2DM. The better performance in the GTT indicates improved insulin sensitivity, which is corroborated by the tendency to lower levels of insulin, and the lower hemoglobin glycation levels indicate a lower glycemic burden over time, despite the finding that the glucose levels remained elevated.

CMS121 was developed based on the flavonoid fisetin and has been found to be more effective than fisetin using in vitro ferroptosis and inflammation models [35,78], as well as in mouse AD and aging models [11,38,39]. These results suggested that CMS121 might also be beneficial in other disease models where fisetin had shown positive effects, such as diabetes. In the streptozotocin-induced model of type 1 diabetes, fisetin improved blood glucose, plasma insulin, and glycosylated hemoglobin levels [46,79]. Additionally, fisetin prevented cataract formation in diabetic mice [80], and reversed changes in the liver and kidney, reestablishing the activity of enzymes of carbohydrate metabolism. The restoration of enzyme activities is a potential mechanism contributing to better glucose homeostasis [46,79]. Since CMS121 shares many pharmacological properties with fisetin, we thought it was worth investigating if it also shares some of the same protective mechanisms for improved glucose homeostasis.

CMS121 is a geroneuroprotector that can activate AMPK, leading to decreased fatty acid synthesis [11]. Additionally, CMS121 is a direct fatty acid synthase (FASN) inhibitor [38]. This evidence supports the idea that the lower weight gain, decrease in lipids in blood and liver, and lower fatty acid metabolites in urine are dependent on the ability of CMS121 to suppress fatty acid synthesis.

Excess fuel and lipids can lead to cellular lipotoxicity [47], which was clearly seen in the livers of the control db/db mice, which displayed a strong pro-inflammatory profile. The CMS121 diet alleviated this inflammatory status, as evidenced by lower levels of p-NF-κB and inflammatory cytokines, such as IL-18 and CRP. The anti-inflammatory properties of CMS121 were previously observed in vitro and in a mouse model of AD, where CMS121 normalized the elevated levels of eicosanoids and decreased inflammatory markers, and lipid peroxidation end products [38]. These anti-inflammatory effects were also seen in rapidly aging SAMP8 mice [39]. FASN is crucial for diet-induced activation of macrophages [81], and inhibition of FASN prevents liver damage by reducing liver fat, inflammatory markers, and fibrotic scar tissue [82]. Therefore, it is likely that at least part of CMS121’s hepatic anti-inflammatory effects are also linked to its inhibition of FASN.

Similar to CMS121, metformin is also a geroneuroprotector and an AMPK activator, which can reduce hepatic glucose production by limiting the expression of gluconeogenic genes in the liver [83]. As an energy sensor, AMPK regulates energy balance, but has also been reported to protect against lipid-induced inflammation [84]. AMPK activation was proposed as the mechanism responsible for the decrease in hepatic lipotoxicity in db/db mice [49]. Thus, the reduced inflammatory state caused by the CMS121 diet may also be linked to AMPK activation, possibly in combination with the inhibition of FASN, which remains to be confirmed.

### 3.2. Renal Protection

As noted, CMS121 was designed to target inflammation and neurodegenerative diseases and has anti-ferroptotic activity [10]. Recent studies have emphasized the role of ferroptosis in kidney diseases [85]. Due to the link between kidney disease and neurological dysfunction [86], and the interplay between brain and kidney inflammation [87], we also investigated whether CMS121 would be suitable for kidney protection in the context of T2DM.

Diabetic nephropathy is a serious microvascular complication of diabetes that, due to hyperglycemia, progresses from low-grade renal inflammation to renal fibrosis, sclerosis, and end-stage renal disease [88]. Indicators of renal decline include functional measures such as glomerular filtration rate, as well as markers of glomerular integrity (albumin and clusterin) and of proximal (KIM1) and distal tubule (NGAL) damage in the urine [89,90]. The CMS121 diet greatly reduced urine albumin and clusterin levels in the db/db mice, suggesting improved glomerular filtration. While the low levels of KIM1 in the urine of the db/db mice indicated that the proximal tubules were less affected in this mouse model, the CMS121 diet reduced the dramatic increase in urinary NGAL, indicating strong protection of the distal tubules. Activated mesangial cells produce αSMA and collagen I, markers of kidney fibrosis [91]. The CMS121 diet decreased collagen I levels in the kidneys of the db/db mice but did not change the elevated levels of αSMA, suggesting partial protection from kidney fibrosis.

The urine metabolomic study further supported the idea that the CMS121 diet improved kidney function in the db/db mice. Positive changes in several urine metabolites linked to kidney health were seen. For example, the depleted levels of adenosine, an important mediator of renal blood flow [69,70], were partially restored while the increases in 2′,3′-cAMP and 2′,3′-cGMP, markers of kidney stress [71], were reduced by the CMS121 diet. Decreased levels of the known renal toxins, 5-oxoproline, indoxyl sulfate, and 3-hydroxykynurenine [61,62,63], in the urine of the db/db mice fed CMS121 also suggested protective effects. In addition, elevated levels of N-acetylalanine [68] and glycolate [67] in the urine of db/db mice are also indicative of kidney damage and loss of function. The CMS121 diet also reversed the alterations seen in these two metabolites in the db/db mice.

The protective effects of the CMS121 diet on the kidney were seen previously in aging, senescence-accelerated SAMP8 mice [39]. In this model, CMS121 in the diet reduced several markers of inflammation, including iNOS, NF-κB, and caspase 1. In addition, CMS121 modulated the AMPK, MAPK, and mTOR pathways, leading to improvement in several markers of kidney damage. Although we did not directly examine inflammation markers, CMS121 may also protect against kidney damage in db/db mice by acting as an anti-inflammatory agent as suggested by the effects of CMS121 on kidney NOX4 levels. The possible involvement of AMPK, MAPK, and mTOR pathways in the protective effects of CMS121 against diabetic nephropathy is an interesting topic for future study.

In the context of T2DM, uncontrolled vascular tone can damage mesangial cells, further impairing renal filtration [92]. Renal proximal tubular arginase plays an important role in the kidney aging process [93] and also contributes to vascular dysfunction in apolipoprotein E deficient mice, leading to BBB leakage and neuroinflammation, which has been associated with endothelial dysfunction in T2DM [94]. The phenotypes induced by arginase can be reversed by inhibitors [95]. Interestingly, fisetin has a clear inhibitory effect on arginase [96], and also ameliorates atherosclerosis by regulating PCSK9 and LOX-1 in apolipoprotein E deficient mice [97]. Since CMS121 was designed based on the natural flavonol fisetin, we can speculate that a possible arginase inhibitory effect could contribute to the renoprotective effects of CMS121.

Our study also assessed the effects of the CMS121 diet on mitochondrial markers in db/db mouse kidneys. Previous research has shown that treatment with the β2-adrenergic receptor agonist formoterol can revert increased levels of ETC complexes in the db/db mouse kidney. Our study found that the CMS121 diet also restored normal levels of respiratory complexes I and III as well as VDAC in db/db mouse kidneys. Further investigation is required to understand the underlying mechanisms and the role of transcriptional control of mitochondrial proteins, such as PGC1α, PPARy, TFAM, and Nrf1, in these actions of CMS121. The CMS121 diet was also shown to alleviate increased levels of numerous mitochondrial genes encoding ETC proteins in aged SAMP8 mice brain [11]. These studies suggested that activation of AMPK leading to elevated acetyl-coenzyme A and inhibition of lipid synthesis may provide the mechanism underlying this effect.

It is noteworthy that changes in mitochondrial function are reported to precede histological and biochemical changes related to kidney damage, suggesting that mitochondrial dysfunction may contribute to such damage, particularly in the context of diabetes [98]. In the early stages of diabetic kidney disease in animal models, metabolic activity in tubular cells has been shown to increase [99], with reports confirming increased respiratory complex activity [100]. Our findings are consistent with these reports, showing increased levels of mitochondrial proteins in db/db mice, which were reversed by CMS121 treatment. Elevated production of reactive oxygen species has also been proposed as a potential mechanism for disrupted mitochondrial respiration [101].

Increased NOX4 levels have been associated with CKD and contribute to oxidative damage and diabetic glomerular dysfunction [102]. Under stress, NOX4 can migrate to the mitochondria, leading to increased ROS production, especially by modulating complex I activity [102]. High fumarate levels have also been linked to a renal phenotype in phospholipase A2 receptor autoimmunity with depleted FH activity, while restoration of FH improved renal function [103]. A clinical study showed that increased serum and urine fumarate may be linked to oxidative stress and CKD progression [104]. Inhibition or deletion of NOX4 has been shown to slow the progression of diabetic kidney disease [74]. Induction of renal NOX4 elevates fumarate levels by inhibiting FH, which was correlated with a NOX4-dependent generation of H_2_O_2_ [74]. The CMS121-induced decrease in renal NOX4 and MDA levels correlated with lower fumarate levels in the urine. Therefore, we can speculate the NOX4/fumarate mechanism may also contribute to oxidative damage and kidney dysfunction in db/db mice, which is another way that CMS121 might mitigate kidney damage in these mice.

In summary, the evidence suggests that CMS121 can improve kidney function in the context of T2DM. This is indicated by improved urine markers of kidney damage, the results from the urine metabolomics study, the normalization of mitochondrial protein levels, and the reduction of renal NOX4 expression, MDA levels, and urinary fumarate. Further research is needed to fully understand the mechanism of CMS121-dependent renal protection, as well as the factors that control ETC proteins, and the connections between fumarate, NOX4 induction, and renal protection.

## 4. Material and Methods

### 4.1. Study Design

The objective of this study was to evaluate the effects of CMS121 on diabetes status, liver inflammation, and kidney fibrosis markers in the db/db mouse background [41]. Mice lacking the leptin receptor (db/db) on the C57BL/6J genetic background become obese and develop T2DM in the first weeks of life. Liver inflammation and CKD are also important consequences of T2DM as the disease progresses, with CKD being a main cause of fatality in humans [2,3].

A timeline and the endpoints analyzed are presented in Figure 1A. At 5 weeks of age, male db/db mice and untreated wildtype (WT) mice (C57BL/6J) were fed a standard rodent diet (LabDiet 5015 (25% fat), TestDiet, Richmond, IN) with or without CMS121 for 6 months. Mice (12 per group) were assigned randomly to treatment diet (db/db + 121), and control group (db/db). WT mice were also evaluated and used as a reference group to highlight the db/db phenotype. The food intake corresponds to an average consumption of 9.4 mg/kg/day CMS121 during the first 17 weeks of treatment (200 ppm) and 18.8 mg/kg/day CMS121 for weeks 18–24 (400 ppm), based on the measurement of the overall food consumption of 3 mice housed in each cage. The dose of CMS121 was based on previous work where it was found to be effective as a geroprotector in mouse models of aging and AD [11,38,39]. Metabolic testing and data collection were performed blinded to the researcher. At different time points, and as indicated in the results section, urine and blood were collected for glucose, lipids, and albumin evaluation. Body mass and metabolic status were evaluated at the 13th and 15th weeks of treatment, respectively. After 6 months of treatment, animals were sacrificed, and blood, liver, and kidney collected for further analyses.

All studies were carried out in accordance with the recommendations in the Guide for the Care and Use of Laboratory Animals of the National Institute of Health. The protocol was approved by the Animal Care and Use Committee of the Salk Institute for Biological Studies. 

### 4.2. Body Mass and Metabolic Evaluation

Echo-MRI analysis was performed at the 13th treatment week to evaluate body mass using an EchoMRI 100 apparatus (Echo Medical Systems, Houston, TX, USA). At the 15th week of treatment, animals were placed for 5 days in a metabolic cage system apparatus (LabMaster, TSE-Systems Inc., Chesterfield, MO, USA) equipped to detect indirect calorimetry, measure food and water intake, and monitor activity. Metabolic parameters, such as oxygen consumption (VO_2_), carbon dioxide production (VCO_2_), respiratory exchange ratio (RER), and energy expenditure, were obtained, and data analyzed with CalR 1.1 software [105].

### 4.3. Biochemical Analyses

For urine collection, mice were individually placed in metabolic cages for 24 h, and urine collected at 12 and 24 h and pooled.

Urine proteins were extracted by the acid/acetone precipitation method [106]. Briefly, urine samples were centrifuged at 3000× *g* for 10 min to remove any particulate material. The supernatant was incubated with 6% trichloroacetic acid for 2 h at 4 °C. The sample was centrifuged at 14,000× *g* for 15 min at 4 °C. The resulting pellet was washed with cold acetone, and the centrifugation was repeated (14,000× *g*, 15 min, 4 °C). The supernatant was discarded, while the pellet was air dried, and the solid pellet diluted in Western blot sample buffer (25 mM Tris, pH 8.0, 2% SDS, 25 mM 2-mercaptoethanol, 1 mM Na_3_VO_4_).

Tail blood was collected for assay of glucose, as performed with the enzymatic Infinity Glucose Hexokinase Liquid Stable Reagent (Thermo Scientific, Middletown, VA, USA) or by Accu-Check Aviva test strips (Roche, Indianapolis, IN, USA). Hemoglobin A1c (HbA1c) was measured by an enzymatic assay and insulin by ELISA, both from CrystalChem (Elk Grove Village, IL, USA). TG and plasma cholesterol were assayed by commercial kits (Pointe Scientific, Canton, MI, USA). Liver cholesterol was assayed with a fluorometric assay (Cell Biolabs, San Diego, CA, USA).

For the GTT, mice were deprived of food overnight, and a glucose solution was given by gavage (0.5 g/kg), as previously described [107]. Blood glucose was sampled at 0, 30, 60, 90, and 120 min by tail vein puncture and evaluated by Accu-Check Aviva test strips (Roche, Indianapolis, IN, USA).

At the end of the experiment, mice were anesthetized, and their blood collected by cardiac puncture. Red blood cells and plasma were separated by using Microvette CB 300 K2E blood separation tubes (Sarstead, Nübrecht, Germany). After perfusion with PBS, liver and kidneys were removed and stored at −80 °C until further use.

### 4.4. Western Blotting

Liver and kidney samples were mechanically homogenized with TissueRuptor (Qiagen, Hilden, Germany) at a 1:5 w/vol in mitochondria buffer (Hepes 5 mM, EGTA 1 mM, mannitol 220 mM, sucrose 70 mM) with added Roche (Indianapolis, IN, USA) protease and phosphatase inhibitors cocktail. After centrifugation (800× *g*, 10 min, 4 °C), the pellet was washed with PBS/0.1% NP40, centrifuged again, and then used as the nuclear fraction. The supernatant was centrifuged at 10,000× *g* for 10 min at 4 °C, and the pellet was considered the mitochondrial fraction, while the supernatant was used as the cytosolic fraction. Samples were diluted in Western blot sample buffer. Protein was assayed by the BCA method (Invitrogen, Waltham, MA, USA).

Samples were analyzed by SDS-PAGE using 10, 12, or 15% gels, according to the molecular weight of the target protein (Criterion XT Precast Bis-Tris Gels, Bio-Rad, Hercules, CA, USA). Proteins were transferred to polyvinylidene fluoride membranes and probed with the desired primary antibody. Stain-free gels were used to quantify proteins (Bio-Rad). Horseradish peroxidase-conjugated secondary antibodies were diluted 1/5000 in 2% skim milk in tris buffered saline/0.1% Tween 20 prior to use. Images were obtained by the Chemidoc MP Imaging System (Bio-Rad) and quantified with the ImageJ 1.54d software (https://imagej.nih.gov/ij/, accessed on 30 March 2023).

The following antibodies were used from Cell Signaling p-NF-κB (phospho-p65 Ser536; #S3033), NFκB (#8242), IL-1β (#63124), caspase 1 (#4199), caspase 3 (9661), albumin (#4929), α-smooth muscle actin (αSMA, #19245), TOMM20 (#42406), and VDAC (#4866); collagen I (PA5-95137) was from Invitrogen (Waltham, MA); clusterin alpha (#SC-6419) was from Santa Cruz; CRP (#ab65842), KIM1/TIM1 (#ab233720), NGAL (ab216462), and MDA (#ab27642) were from Abcam (Cambridge, UK); and IL-18 (#210-401-323) was obtained from Rockland (Royersford, PA, USA). Mitochondrial ETC markers (#ab110413) were obtained from Abcam: complex I (CI, NDUFB8), CII (SDHB), CIII (UQCRC2), CV (ATP5A).

### 4.5. Metabolomic Analyses of Urine Samples

Metabolomic analysis was performed at Metabolon with urine samples collected at the 12th treatment week, using 6 animals in each group. The sample preparation and initial analysis were done by Metabolon, as previously published [108], except that quality control was performed with urine samples. We then recalculated the fold changes and performed the statistical analyses as follows. Datasets with 3 or more missing values were excluded from the analysis, as well as outliers. The outliers were identified using the interquartile range (IQR) of data, which is the range between the first (Q1) and the third (Q3) quartiles (IQR = Q3 − Q1). The data points which fell below Q1 − 1.5 × IQR or above Q3 + 1.5 × IQR were defined as outliers. After filtering out 54 metabolites, a total of 700 metabolites were identified. Forty-eight metabolites presented differences between untreated and CMS121-treated db/db mice. Pairwise comparison was performed by the Wilcoxon rank-sum test and considered significant when *p* < 0.05.

### 4.6. Statistical Analyses

One-way ANOVA was used to detect mean changes, and differences between db/db and db/db + 121 were evaluated by Holm–Sidak post-hoc test, when appropriate. Unless otherwise specified, the data are presented as mean ± standard deviation, and sample size is indicated in the figure legends.

## 5. Conclusions

The evidence presented shows that the CMS121 diet improves glucose and lipid metabolism, as demonstrated by: (a) reduced levels of short and medium acylcarnitine intermediates and other lipid metabolites in urine, (b) a lipid-lowering effect in blood and liver, (c) improved glucose tolerance and reductions in HbA1c and insulin, and (d) decreased liver inflammation. These results in the db/db model of obesity and diabetes warrant further investigation of CMS121 for metabolic improvement in additional models of metabolic dysfunction.

Additionally, the CMS121 diet shows promise for kidney protection through: (a) improved markers of renal filtration and kidney damage, (b) positive alterations in urinary metabolites indicative of a renal protection mechanism, (c) restoration of mitochondrial proteins, and (d) decreased expression of NOX4, MDA and the levels of fumarate. Further exploration of the signaling pathways activated by CMS121 is needed to better understand the underlying protective mechanisms.

## Data Availability

The data presented in this study are available in the article and in the Supplementary Material S1.

## Supplementary Materials

The following supporting information can be downloaded at: https://www.mdpi.com/article/10.3390/ijms24076828/s1.
